# Ethnicity, Progressive Keratoconus, and Outcomes after Corneal Cross-Linking in Southern Israel

**DOI:** 10.3390/life13122294

**Published:** 2023-12-01

**Authors:** Jacob A. Yaffe, Ran Matlov Kormas, Boris E. Malyugin, Matthew Boyko, Raimo Tuuminen, Boris Knyazer

**Affiliations:** 1 Department of Ophthalmology, Soroka University Medical Center, Beer Sheva 8457108, Israel; ran_kormas@yahoo.com (R.M.K.); knyazer@bgu.ac.il (B.K.); 2 Faculty of Health Science, Ben Gurion University of the Negev, Beer Sheva 8443944, Israel; 3 S. Fyodorov Eye Microsurgery Federal Institution, Moscow 127486, Russia; 4 Department of Ophthalmology, A. Yevdokimov Moscow State University of Medicine and Dentistry, Moscow 127473, Russia; 5 Department of Anesthesiology and Critical Care, Soroka University Medical Center, Ben Gurion University of the Negev, Beer Sheva 8457108, Israel; 6 Helsinki Retina Research Group, Faculty of Medicine, University of Helsinki, 00014 Helsinki, Finland; 7 Eye Centre, Kymenlaakso Central Hospital, 48210 Kotka, Finland

**Keywords:** epidemiology, ectasia, Bedouin, Jewish, K_max_

## Abstract

Purpose: To assess clinical outcomes of corneal cross-linking (CXL) intervention in a population diagnosed with progressive keratoconus. Methods: This single-center retrospective cohort study included consecutive patients who underwent standard CXL or accelerated CXL for progressive keratoconus at a major teaching hospital in southern Israel between January 2015 and December 2019. Patients’ medical files were reviewed, and pre-operative and post-operative data regarding demographics and clinical and tomographic characteristics were extracted and analyzed. Results: This study included 166 patients (representing 198 eyes), out of which 98 patients (123 eyes) were ethnically Bedouin, and 68 patients (75 eyes) were ethnically Jewish. Overall, 126 patients (144 eyes) had a follow-up of at least 12 months (16.84 ± 5.76). The mean patient age was 20.62 ± 7.1 years old. There were significant baseline differences between the two ethnic groups in best-corrected visual acuity (BCVA; *p* < 0.001), uncorrected visual acuity (UCVA; *p* < 0.001), mean keratometry (*p* = 0.028), and corneal thickness (*p* < 0.001). Significant changes in BCVA, UCVA, and pachymetry parameters within each group were found after 12 months. Negative binomial regression analysis showed a maximal keratometry below 55D (RR = 1.247, *p <* 0.001), and a standard CXL procedure (RR = 1.147, *p* = 0.041) are significantly related to the stability of KC after 12 months. However, the effect size of the origin of patients is negligible (RR = 1.047, *p* = 0.47). Conclusions: In this study, the Bedouin population suffered from more progressive keratoconus when compared to the Jewish population. CXL was significantly effective in improving BCVA and UCVA in both groups after 12 months of follow-up. The effect size of the origin of patients on the stability of KC was found to be negligible.

## 1. Introduction

Keratoconus (KC) is a common bilateral non-inflammatory ectatic corneal disorder characterized by stromal thinning, irregular astigmatism of the cornea, and reduced visual acuity (VA) [1]. The disease is multifactorial with many risk factors, including familial, environmental, and regional factors, as well as atopy and eye rubbing. Incipient stages of the disease can be asymptomatic and are often diagnosed only by revealing the changes in the corneal parameters by corneal tomography [2]. This method is considered the gold standard for diagnosis and monitoring the disease. Tomographic parameters reveal steeping of the anterior and posterior surface and the corneal thickness (pachymetry) [3,4]. As the disease progresses, cone-shaped cornea may be visible at the slit lamp [3], VA deteriorates, and patients may require penetrating keratoplasty. Hence, stabilization of the disease in its early stages is crucial.

Corneal cross-linking (CXL) has improved the treatment of ectatic corneal disorders, particularly KC [5]. CXL forms chemical bonds among collagen fibrils based on an interaction between ultra-violet A irradiation at a wavelength of 370 nm and riboflavin [6,7]. Case–control studies showed a significant decrease in the disease progression after CXL treatment, particularly presented by improvements in keratometry values and in VA in progressive disease cases [8].

Soroka University Medical Center (SUMC) is a major academic hospital in the Negev area in southern Israel and serves both Jewish (approximately 60%) and Bedouin (approximately 36%) populations [9]. The prevalence of KC in the Middle East has been found to be about 2–5% [10,11,12]. In Israel, KC has been associated with eye rubbing, a family history of KC, and lower socioeconomic status [13]. The Bedouin population constitutes a genetically homogeneous minority marked by a high prevalence of consanguineous marriages. Furthermore, this demographic group is characterized by low socioeconomic status, heightened susceptibility to dust exposure, and a comparatively diminished accessibility and awareness of screening tests for ocular diseases in contrast to the Jewish population [14]. Chorney and colleagues have found the socioeconomically challenged Bedouin minority to be at risk for health complications such as diabetic retinopathy [15]. In this study, we focused on the corneal differences between SUMC’s ethnically diverse KC patients.

A previous meta-analysis population study on the natural progression of KC has found that ethnicity significantly affects corneal characteristics such as topography, VA, and pachymetry [16]. Fedi et al. found maximum keratometry (K_max_) significantly steeper among Middle Eastern populations when compared to European and East Asian populations [16]. This study seeks to compare these corneal characteristics between the populations we encounter in SUMC after an intervention such as a CXL procedure. We expect that assessment of the differences between two ethnic groups with different approaches towards health and diseases [17] in terms of the consequences of KC treatment will elucidate the gravity of the disease and will help us understand treatment efficiency for each population.

## 2. Materials and Methods

### 2.1. Participants

This single-center retrospective cohort study included consecutive patients who underwent standard CXL (S-CXL) or accelerated CXL (A-CXL) for progressive KC at the Department of Ophthalmology at a major teaching hospital in southern Israel, (SUMC) between January 2015 and December 2019 and signed a consent form.

Exclusion criteria were a history of previous ocular surgery, history of other corneal diseases (herpes keratitis, recurrent erosions, or corneal inflammation), autoimmune or rheumatic diseases, diabetes mellitus, pregnancy or lactation, and sensitivity to riboflavin or any other substance used in CXL procedure. Patients were instructed to avoid eye rubbing prior to and during every follow-up. Patients with signs or symptoms of vernal kerato-conjunctivitis were treated appropriately to reduce the chances of eye rubbing.

CXL was performed after a diagnosis of Progressive KC. Progressive KC was considered as an increase of 1.50 diopters (D) in mean keratometric value and/or 1.00 D increase in K_max_, and/or a decrease of 5.0% in central corneal thickness at two consecutive evaluations within the last 12 months by Scheimpflug-based corneal tomography (Pentacam HR; Oculus Optikgeräte, Wetzlar, Germany) [18,19,20,21,22].

Statistical comparison was applied only to patients with available data after at least 12 months of follow-up post-CXL procedure.

### 2.2. Data Collection

Medical files of all patients were reviewed, and the following data were extracted: age, gender, ethnicity, date regarding CXL procedure, minimal corneal thickness (MCT), anterior/posterior mean keratometric power, anterior/posterior flat keratometric power, anterior/posterior steep keratometric power, maximum keratometric power, and uncorrected/corrected distance visual acuity. Keratometry and total corneal thickness were measured with Pentacam. Data regarding corneal thickness, keratometric power, and VA were measured before and after CXL was performed.

### 2.3. Main Outcomes Measures

The primary outcome in this study was the change of K_max_ in the two study populations prior to CXL procedure and at the most recent visit (12 to 34 months after CXL). Secondary outcomes in this study were VA measures (uncorrected/corrected distance visual acuity) and corneal topography data of both anterior and posterior segments of the cornea: average keratometry value (Kmean), steep keratometry (Ksteep) and flat keratometry (Kflat), corneal astigmatism, and corneal pachymetry prior to CXL at the most recent visit (12 to 34 months after CXL), as well as stabilization of KC disease. KC stabilization after the CXL procedure was defined as either a decrease in maximum anterior keratometry (K_max_) or as an increase in K_max_ below 1.5 D in the follow-up visit 12 months after the CXL procedure. In cases of suspected progression, a repeat exam including imaging was performed to confirm the diagnosis. The occurrence of any adverse events throughout the study period was recorded.

### 2.4. Surgical Technique

Following topical anesthesia with 0.4% benoxinate hydrochloride drops, MCT was confirmed by ultrasound pachymeter (PachPen; Accutome, Malvern, PA, USA). Following the removal of the central 8 mm of epithelium, the MCT was re-measured to ensure MCT was above 400 μm. Randomly, either standard CXL (S-CXL) or A-CXL was then performed.

Briefly, iso-osmolar 0.1% riboflavin solution (Medio-Cross 0.1%; Peschke Meditrade, Huenenberg, Switzerland) was instilled every 2 min for 20 min. Adequate riboflavin penetration was confirmed by appropriate flare in the anterior chamber. The cornea was then continuously irradiated at 365 nm with either an intensity of 3 mW/cm^2^ for 30 min (S-CXL, total fluence 5.4 J/cm^2^) or an intensity of 9 mW/cm^2^ for 10 min (A-CXL, total fluence 5.4 J/cm^2^) using a commercially available device (LightLink-CXL; LightMed, San Clemente, CA, USA). The patient was instructed to fixate on the light source and adequate centration was constantly monitored by one of the two surgeons (BK or RMK). In cases where MCT was less than 400 μm, contact lens-assisted CXL modification was performed using a method similar to the method which was performed by Matlov Kormas and colleagues [23].

### 2.5. Patients Follow-Up

The patients were prescribed topical ofloxacin 0.3% four times a day for a period of 10 to 12 days and topical dexamethasone 0.1% for a total of 1 month with gradual tapering. Patients were advised to use preservative-free artificial tears as needed. Follow-up visits were routinely performed 1 day, 1 week, and 1, 6, and 12 months following treatment. The bandage contact lens was removed at the 1-week visit after full re-epithelialization of the cornea and corneal tomography, UCVA and BCVA were reassessed at the 12-month visit.

### 2.6. Statistical Analysis

Clinical parameters were tabulated and analyzed using SPSS (version 23; IBM, Armonk, NY, USA). For the analysis of demographical and clinical characteristics, a *t*-test was used for normally distributed variables and the Mann–Whitney test for continuous variables departing from normal distribution. To compare continuous parameters before and after treatment, we used paired *t*-test and Wilcoxon test for normally distributed variables and other factors, respectively. A negative binomial regression model was performed to estimate an effect independent of various factors while adjusting to potential confounders, e.g., origin, K_max_ below 55D at baseline, MCT above 450 μm at baseline, standard CXL procedure and Vernal Keratoconjunctivitis per history. We used generalized estimating equations to adjust for clusters created by patients who were treated for both eyes.

## 3. Results

We included 166 patients (representing 198 eyes), of which 98 patients (123 eyes) belonged ethnically to the Bedouin group and 68 patients (75 eyes) belonged ethnically to the Jewish group. In total, 126 patients (144 eyes) had a follow-up of at least 12 months (16.84 ± 5.76). The mean age ± std. deviation of patients was 20.62 ± 7.1 years old, and 62.6% had been identified as male in their patient files. Table 1 shows patient demographic and clinical characteristics stratified by ethnic groups.

There were significant baseline differences between the two ethnical groups in best-corrected visual acuity (BCVA), uncorrected visual acuity (UCVA), mean keratometry, and corneal thickness. A deeper analysis of the corneal thickness thinnest point showed a significant difference between the groups in different levels, while the thickness tends to be significantly higher in the Jewish group (see Table 1).

The intra-group comparison showed no significant difference in K_max_ and K_mean_ after 12 months when compared to the baseline. However, there was a significant difference in BCVA, UCVA, and corneal thickness (central, apical, and minimal point) after 12 months.

We found a significant difference between the Jewish and Bedouin populations in the Negev area of southern Israel in corneal thickness prior to CXL interventional procedure (see Table 1). The differences in corneal thickness were also significant between each group before and after CXL was performed (see Table 2).

Moreover, we found that corneal thickness was significantly higher among the Jewish group than in the Bedouin group (see Table 1).

CXL procedure has been found significantly effective in improving UCVA and BCVA in both groups after 12 months of follow-up (see Table 2). However, no significant findings were found in comparison between the groups in the mean change of corneal characteristics (see Table 2).

**Figure 1:**
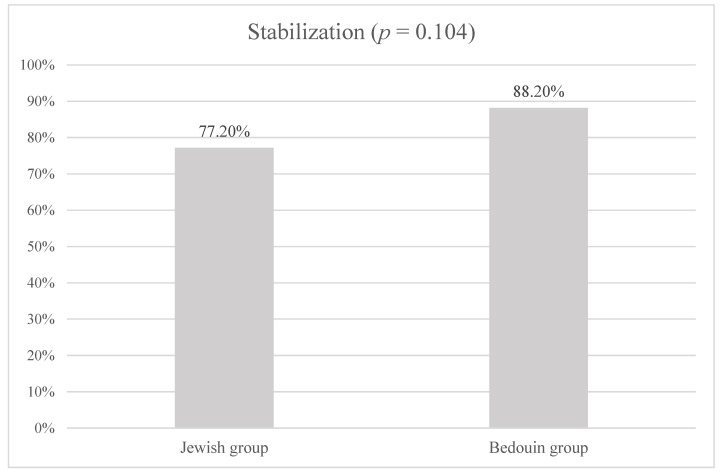
Stabilization (percentage) by ethnicity.

Stability was defined as an increase in less than 1.5 D or a decrease in K_max_ after 12 months after the CXL procedure was performed. This study has found that the stability was 77.2% in the Jewish group and 88.2% in the Bedouin group (see Figure 1).

A negative binomial regression analysis (see Table 3) showed K_max_ below 55 D (RR = 1.247, *p* < 0.001) and a S-CXL procedure (RR = 1.147, *p* = 0.041) are significantly related to the stability of KC after 12 months of follow-up. However, the effect size of the origin of patients is negligible (RR = 1.047, *p* = 0.472 for Jewish origin).

### Safety

No cases were aborted following epithelium removal due to insufficient minimum corneal thickness, indicating that planning was adequate in all cases. All epithelial erosions were completely healed within 1 week. There were no cases of postoperative keratitis or corneal melting. Clinically significant stromal haze occurred in 4 cases in the Jewish group and 5 eyes in the Bedouin group at 1 month following treatment and was treated with an increased frequency (every 2 h) of topical dexamethasone 0.1% with complete resolution by 3 months.

## 4. Discussion

KC is a relatively common multifactorial disease. Even though previous studies suggested both genetic and environmental risk factors [24,25], the etiology of KC disease remains unknown. KC has been associated with eye rubbing, atopy, floppy eyelid syndrome, pregnancy, and thyroid hormone disturbances [25]. Studies regarding eye rubbing, for example, showed a reduction of 18.4% in epithelial thickness immediately after rubbing [26], and some even found eye rubbing to be the most significant risk factor for KC [27]. Atopy has also been associated with KC [28], though some authors suggested that the association is indirect only via itch which leads to eye rubbing [27]. Kaya and colleagues showed that KC patients with atopy had a steeper and thinner ectatic cornea [29]. Studies evaluating the effect of pregnancy showed that KC progressed during the pregnancy period and continued to progress during the post-partum period [30].

The tendency of corneal thickness to be higher among the Jewish group than the Bedouin group (see Table 1) can be viewed through the risk factors associated with KC.

Data regarding eye rubbing can be complicated to compare between groups; however, its association with KC development and progression can be compared indirectly through atopy [27]. Regarding atopy, asthma, for example, has been found to be more prevalent among low socioeconomic populations in 63.0% of studies reviewing the issue [31]. Being a socioeconomically challenged population in southern Israel means also significant exposure to dust and sand. Thus, some corneal characteristics of the Bedouin group can be explained by these risk factors. Since KC has been found to progress during pregnancy, an extremely high birth rate among the Bedouin population might also contribute to the progression of KC among this population [30,32,33]. This finding could possibly explain the higher number of Bedouin women (46.0%) in comparison to Jewish women (22.1%) in our study group (see Table 1).

In addition to corneal thickness, other baseline corneal and clinical parameters were significantly inferior in the Bedouin group than in the Jewish group (e.g., UCVA, BCVA, and K_mean_). These parameters can emphasize the fact that Bedouin patients have more progressed levels of KC. Studies regarding genetic factors and consanguinity, a prevalent phenomenon among the Bedouin population in the Negev [34] have found that children of consanguineous parents have a four-fold risk of KC when compared to children of unrelated parents [35].

Positive family history of KC has also been found significantly higher among the Bedouin group (see Table 1); a factor considered to increase risk for KC [11] can help explain the inferior corneal condition of this group. Genetic etiology demonstrated by familial inheritance, discordance between dizygotic twins, and its association with other known genetic disorders have been extensively discussed by multiple research groups. Reports about family history of KC have shown proportions ranging between 5% to 10% and even up to 23% [36,37]. Several genetic loci were found to be associated with KC, and several genes were found to be associated with KC symptoms [37]. Due to the retrospective design of our study, this issue was not addressed.

Studies regarding compliance among the Bedouin population in a variety of diseases and disorders (e.g., cardiovascular disorders [38], diabetes mellitus [39], and genetic screening tests [40]) have concluded that compliance in this population is lower than in the Jewish population. Lower mean levels of corneal characteristics among the Bedouin group (see Table 2) can be explained via this fact also.

CXL procedure has been found to be significantly effective in improving UCVA and BCVA in both groups after 12 months of follow-up with no significant difference between the groups. Our multivariate analysis showed the effect size of the origin of the patients is negligible. These findings implicate that the CXL procedure is effective in halting the progression of KC regardless of ethnic group or level of KC. Moreover, stabilization rates were found to be similar in both groups, confirming this conclusion.

The corneal differences we report in this study raise questions regarding steps that possibly can be taken towards the Bedouin minority for prevention, diagnosis, and treatment of KC (e.g., screening tests). First, raising awareness for such pathology and its ramifications may lead to better collaboration and early diagnosis. A clear and plain explanation of the disease causes, prevention, treatment, and routine follow-up, both clinically and diagnostically, may increase both awareness and response to routine check-ups. Secondly, improving the availability of ophthalmology services for the Bedouin population and performing routine examinations may identify patients with KC in the early stages. For example, retinoscopy is a simple screening test that can implicate the existence of KC. Al-Mahrouqi and colleagues have found that retinoscopy can serve as a sensitive and reliable test for the detection of KC even in early stages [41]. Another example of a screening tool is imaging. Corneal and epithelial thickness maps and patterns, in addition to corneal topography or tomography, can be used to improve the screening of KC disease with a sensitivity of 97.8% [42].

Regarding stability, our multivariate analysis has found a connection between preoperative maximal keratometry less than 55 D to stabilization of CXL after 12 months of follow-up (see Table 3). Koller and colleagues also found higher K_max_ a significant risk factor for failure of CXL (K_max_ > 58 D) [43]. Another study showed that patients with K_max_ > 60 D were at higher risk for failure. S-CXL procedure has also been found positively related to stability [44] (see Table 3). This finding is supported by other studies that have found S-CXL associated with higher stabilization rates than A-CXL [45,46]. Regarding differences between the Jewish and Bedouin populations, our multivariate study has found the differences to be negligible (see Table 3), suggesting that the procedure results do not depend on the ethnic origin of the patients but on the preoperative severity of the disease.

A delay in diagnosis among the Bedouin population and the rapid deterioration emphasize that early intervention should be considered. Performance of CXL procedure under the age of 17 years old, was not found significantly different than among patients over 17 years old [47], implicating that early intervention is effective for early stabilization and better progress of corneal parameters [48]. Therefore, we suggest that early intervention should follow the diagnosis with the aim of achieving better rates of disease stability and halt the disease progression in earlier stages among the Bedouin population.

We have found CXL to be effective in halting the progression of KC for at least one year regardless of ethnicity and stage at diagnosis. We have also found significant tomographic and clinical differences between the Jewish and Bedouin populations in southern Israel. These differences can be explained by a delayed diagnosis, cultural differences (e.g., consanguinity), negative influences of some environmental factors (allergy, atopy), and poor compliance to treatment and follow-up. We suggest that health promotion regarding the consequences of untreated KC, screening tests (e.g., refraction, retinoscopy, and corneal topography), and early intervention should take place in the community clinics of the minority population in the Negev.

Considering the Bedouin population in the Negev is a socioeconomically challenged minority with limited access and compliance to healthcare, our conclusions might be relevant to similar minority groups around the world.

## 5. Study Limitations and Future Research

This study does have limitations. As a retrospective study, the conclusions are limited to available data. A future prospective study would allow more control, which could lead to more specific conclusions for each population. In addition, only 72.0% of patients who had undergone the CXL procedure had a follow-up of a least 12 months. Thus, our conclusions could bias the comparison between the groups towards only compliant patients. Future work could ensure a more stringent follow-up protocol. In addition, due to the retrospective design of our study, genetic differences between the groups were not assessed. Identification of the genetic factors of KC disease, specifically between different ethnic groups, could help develop diagnostic tools and therapeutic methods in the future and therefore further research and analysis could help address this issue.

## Figures and Tables

**Table 1:** Demographic and clinical characteristics.

Patient Characteristics	Jewish Group (68 Patients, 75 Eyes)	Bedouin Group (98 Patients, 123 Eyes)	*p*-Value
Demographical characteristics			
Time until follow-up, months	17.55 ± 6.58 (57/75)	16.38 ±5.14 (87/123)	0.256
Mean ± SD (*n*)			
Age, years	23.64 ± 6.90 (75)	18.68 ± 6.60 (123)	<0.001
Mean ± SD (*n*)			
Gender, % (n/N)	77.9% (53/68)	54.0% (53/98)	0.002
Male			
Treated eye, % (n/N)	46.7% (35/75)	49.6% (61/123)	0.770
Right eye			
Family history of KC, % (n/N)	12.1% (8/66)	39.1% (36/92)	<0.001
Yes			
Vernal Keratoconjunctivitis per history, % (n/N)	18.4% (12/65)	29.4% (28/95)	0.138
Yes			
Wearing of glasses, % (n/N)	37.1% (23/62)	37.0% (30/81)	1.000
Yes			
Clinical characteristics			
Type of CXL, % (n/N)			0.013
Standard	32.0% (24/75)	16.3% (20/123)	
Accelerated	68.0% (51/75)	83.7% (103/123)	
UCVA, logMAR	0.59 ± 0.45 (73)	0.89 ± 0.59 (106)	<0.001
Mean ± SD (*n*)			
BCVA, logMAR	0.30 ± 0.21 (73)	0.45 ± 0.24 (107)	<0.001
Mean ± SD (*n*)			
K_max_, D	56.28 ± 6.62 (75)	58.15 ± 8.94 (119)	0.229
Mean ± SD (*n*)			
K_mean_, D	48.34 ± 4.28 (75)	50.24 ± 5.87 (122)	0.028
Mean ± SD (*n*)			
MCT, μm	465.18 ± 46.42 (75)	434.85 ± 49.52 (122)	<0.001
Mean ± SD (*n*)			
MCT, % (n/N)			<0.001
<400 μm	8.0% (6/75)	8.0% (22/122)	
400 μm < Thickness < 450 μm	30.7% (23/75)	41.8% (51/122)	
450 μm < Thickness < 500 μm	30.7% (23/75)	32.0% (39/122)	
>500 μm	30.7% (23/75)	8.2% (10/122)	

Note: N = number of patients; SD = standard deviation; D = diopter; UDVA = uncorrected distance visual acuity; BCVA = best-corrected visual acuity; K = keratometry; MCT = minimum corneal thickness.

**Table 2:** Preoperative and 12 months postoperative clinical parameters by ethnic group.

Clinical Parameters	a. Intra-Group Comparison						b. Inter-Group Comparison (Presented by Mean Change in Clinical Outcomes)		
	Jewish Group (52 Patients, 57 Eyes)		*p*-Value	Bedouin Group (74 Patients, 87 Eyes)		*p*-Value	Jewish Group (52 Patients, 57 Eyes)	Bedouin Group (74 Patients, 87 Eyes)	*p*-Value
	Baseline (N = 57)	>12 Months (N = 57)		Baseline (N = 87)	>12 Months (N = 87)				
UCVA (logMAR) Mean ± sd (*n*) Median Min; Max	0.57 ± 0.44 (55) 0.4 0.00; 2.00	0.44 ± 0.39 (52) 0.26 0.00; 2.00	0.003	0.90 ± 0.59 (76) 0.74 0.00; 3.00	0.62 ± 0.46 (69) 0.5 0.00; 2.00	<0.001	−0.12 ± 0.35 (52) −0.17 −1.00; 1.40	−0.23 ± 0.53 (66) −0.11 −2.00; 0.90	0.560
BCVA (logMAR) Mean ± sd (*n*) Median Min; Max	0.32 ± 0.22 (55) 0.22 0.00; 1.00	0.23 ± 0.21 (52) 0.2 0.00; 1.00	0.008	0.45 ± 0.25 (79) 0.48 0.00; 1.00	0.39 ± 0.36 (79) 0.3 0.00; 2.00	0.003	−0.07 ± 0.21 (50) −0.02 −0.80; 0.60	−0.08 ± 0.29 (73) −0.02 −0.78; 1.00	0.864
K_max_ (D) Mean ± sd (*n*) Median Min; Max	55.80 ± 6.02 (57) 54.1 47.40; 72.00	55.66 ± 6.22 (57) 52.7 45.60; 70.30	0.981	57.15 ± 7.24 (85) 56.1 44.60; 79.60	57.74 ± 7.93 (87) 56.6 46.20; 85.90	0.624	−0.16 ± 2.45 (56) 0 −4.40; 8.60	0.09 ± 2.14 (84) 0.1 −8.60; 7.00	0.973
K1 flat front (D) Mean ± sd (*n*) Median Min; Max	45.87 ± 3.42 (57) 45.3 38.20; 55.70	46.06 ± 3.73 (57) 46 37.70; 56.60	0.257	48.05 ± 5.17 (87) 47.1 36.90; 66.50	48.30 ± 5.24 (87) 47.3 38.80; 65.00	0.566	0.18 ± 2.02 (57) 0 −10.40; 4.70	0.25 ± 1.9 (87) 0 −3.20; 11.70	0.588
K2 steep front (D) Mean ± sd (*n*) Median Min; Max	50.07 ± 4.31 (57) 50.4 42.80; 63.30	49.61 ± 4.26 (57) 49.1 42.20; 65.20	0.238	52.37 ± 5.77 (87) 52.5 40.50; 72.10	52.65 ± 6.11 (87) 51.9 43.50; 70.80	0.979	−0.45 ± 3.01 (57) −0.20 −19.00; 7.60	0.28 ± 3.10 (87) 0 −5.10; 22.90	0.350
K_mean_ front (D) Mean ± sd (*n*) Median Min; Max	47.76 ± 3.62 (57) 47.1 42.20; 59.20	47.74 ± 3.82 (57) 47.3 40.10; 60.60	0.711	50.00 ± 5.31 (87) 49.6 38.70; 69.20	50.40 ± 5.43 (86) 49.5 41.40; 67.20	0.391	−0.02 ± 2.40 (57) 0 −14.00; 6.60	0.33 ± 2.24 (86) 0.1 −3.60; 12.80	0.776
K_mean_ back (D) Mean ± sd (*n*) Median Min; Max	−7.41 ± 0.82 (57) −7.20 −9.90; −6.00	−7.00 ± 0.73 (57) −6.90 −9.60; −5.90	0.479	−7.46 ± 0.92 (85) −7.40 −11.00; −5.60	−7.42 ± 1.39 (85) −7.50 −10.70; 0.60	0.194	−0.02 ± 0.31 (57) 0 −1.30; 1.10	0.08 ± 8.65 (83) 0 −1.70; 6.95	0.747
Astigmatism front (D) Mean ± sd (*n*) Median Min; Max	4.18 ± 2.57 (57) 3.5 1.00; 13.70	3.56 ± 2.17 (57) 2.9 0.40; 9.80	0.125	4.31 ± 2.00 (87) 4 0.10; 11.30	4.28 ± 2.05 (87) 4.2 0.00; 10.20	0.859	−0.61 ± 2.18 (57) −0.30 −8.60; 4.50	−0.02 ± 1.65 (87) 0 −7.10; 4.90	0.327
Astigmatism back (D) Mean ± sd (*n*) Median Min; Max	0.83 ± 0.45 (57) 0.8 0.00; 2.10	0.74 ± 0.43 (57) 0.7 0.10; 2.00	0.166	0.89 ± 0.38 (86) 0.8 0.20; 1.80	0.91 ± 0.82 (87) 0.8 0.00; 6.40	0.104	−0.08 ± 0.37 (57) −0.10 −1.50; 0.60	0.03 ± 0.8 (86) −0.05 −0.90; 5.00	0.932
CCT (μm) Mean ± sd (*n*) Median Min; Max	488.49 ± 44.36 (57) 484 384.00; 574.00	480.64 ± 41.09 (57) 483 385.00; 560.00	0.003	454.77 ± 41.30 (85) 454 356.00; 547.00	440.75 ± 56.84 (85) 442 119.00; 609.00	<0.001	−7.84 ± 19.24 (57) −11.00 −57.00; 40.00	−14.92 ± 45.77 (83) −10.00 −344.00; 93.00	0.639
ACT (μm) Mean ± sd (*n*) Median Min; Max	482.19 ± 44.36 (57) 480 346.00; 542.00	470.45 ± 46.42 (57) 470 355.00; 563.00	<0.001	448.01 ± 41.80 (87) 444 346.00; 542.00	430.47 ± 59.21 (85) 435 108; 599.00	<0.001	−11.73 ± 18.52 (57) −12.00 −60.00; 35.00	−18.04 ± 44.02 (85) −11.00 −333.00; 92.00	0.733
MCT (μm) Mean ± sd (*n*) Median Min; Max	466.86 ± 45.96 (57) 463 338.00; 560.00	453.19 ± 50.12 (57) 460 277.00; 541.00	<0.001	434.70 ± 44.04 (87) 432 339.00; 531.00	418.54 ± 45.69 (86) 423.5 297.00; 517.00	<0.001	−13.67 ± 19.78 (57) −13.00 −83.00; 39.00	−16.6 ± 24.94 (86) −15.00 −98.00; 27.00	0.686
Corneal volume (mm^3^) Mean ± sd (*n*) Median Min; Max	57.32 ± 4.84 (57) 57.4 35.60; 68.70	56.68 ± 4.74 (57) 56.6 34.60; 66.70	0.003	55.98 ± 3.96(85) 55.3 48.30; 68.20	55.11 ± 4.68 (87) 55.2 30.50; 66.00	0.004	−0.63 ± 1.63 (57) −1.00 −4.60; 3.40	−1.02 ± 3.63 (85) −0.60 −27.50; 3.90	0.896

Note: N = number of patients; SD = standard deviation; D = diopter; UDVA = uncorrected distance visual acuity; BCVA = best-corrected visual acuity; K = keratometry; CCT = central corneal thickness; ACT = apical corneal thickness; MCT = minimum corneal thickness. Mean change was calculated as 12+ months post procedure minus at baseline.

**Table 3:** Factors related to KC stability within 12 months after CXL (multivariate analysis results).

Patient Characteristics	RR	*p*-Value	95% Confidence Interval (RR)	
			Lower	Upper
K_max_ < 55 D at baseline	1.24	<0.001	1.11	1.39
MCT > 450 μm at baseline	1.08	0.171	0.96	1.23
Standard CXL procedure	1.14	0.041	1.01	1.31
Jewish origin	1.05	0.472	0.92	1.19
Vernal Keratoconjunctivitis per history	1.06	0.486	0.91	1.23

Note: RR = relative risk; K = keratometry; MCT = minimum corneal thickness; CXL = corneal cross-linking.

## Data Availability

The data presented in this study are available on request from the corresponding author. The data are not publicly available due to privacy issues.
